# Recreational Nitrous Oxide Misuse: Anaesthetic Challenges and Perioperative Complications Including Airway Burns and Neurological Sequelae

**DOI:** 10.7759/cureus.89232

**Published:** 2025-08-01

**Authors:** Nosaiba K Ezzelarab, Tarek Matar

**Affiliations:** 1 Anaesthesia, King's College Hospital NHS Foundation Trust, London, GBR; 2 Neurology, University Hospitals Sussex NHS Foundation Trust, Brighton, GBR

**Keywords:** airway burn, anaesthesia, neurological complications, nitrous oxide, vitamin b12

## Abstract

Recreational use of nitrous oxide (N_2_O) (“hippy crack”) has increased in recent years, with serious complications being reported, including airway cold injuries, neurological disease, and thrombotic events. This review highlights the perioperative implications of N_2_O misuse, with a focus on airway injury, neurological sequelae, and anaesthetic management. Cold burns from direct inhalation can compromise airway safety, while chronic use leads to functional vitamin B12 inactivation, resulting in subacute combined degeneration and peripheral neuropathy. In such patients, intraoperative use of N_2_O may exacerbate existing complications. Clinicians should maintain a high index of suspicion for N_2_O misuse, avoid its administration during anaesthesia, and consider early referral for neurological assessment and substance misuse support.

## Introduction and background

Nitrous oxide (N_2_O), also known as “laughing gas,” is a commonly used anaesthetic agent with well-established analgesic and sedative properties. In clinical practice, it has a favourable safety profile when used under controlled settings. However, recreational misuse of N_2_O has emerged as a growing public health concern in recent years, particularly among adolescents and young adults in Europe and the UK [1]. Readily accessible in whipped cream canisters or industrial cartridges, the gas is often perceived as harmless due to its short-acting euphoric effects and over-the-counter availability [2].

Recent data show an alarming rise in N_2_O-related emergency admissions and long-term complications. The UK’s Office for National Statistics and Public Health England have both reported increases in morbidity and mortality related to recreational use [1]. Of particular concern to anaesthetists are complications such as neurological damage (e.g., subacute combined degeneration (SCD) of the spinal cord), haematological abnormalities, and airway trauma resulting from cryogenic injury.

As the incidence of N_2_O misuse rises, anaesthetists must be aware of the pathophysiological mechanisms, clinical presentations, and potential perioperative hazards associated with this practice. This narrative review outlines the key anaesthetic implications of recreational N_2_O misuse, focusing on airway injuries, neurological complications, and perioperative management considerations.

## Review

Mechanism of action and pathophysiological effects

N_2_O exerts its anaesthetic and analgesic effects primarily through non-competitive inhibition of N-methyl-D-aspartate (NMDA) receptors, which reduces excitatory neurotransmission and promotes central analgesia. It also stimulates the release of endogenous opioids and activates descending inhibitory noradrenergic pathways, enhancing its analgesic action [3].

**Figure 1:**
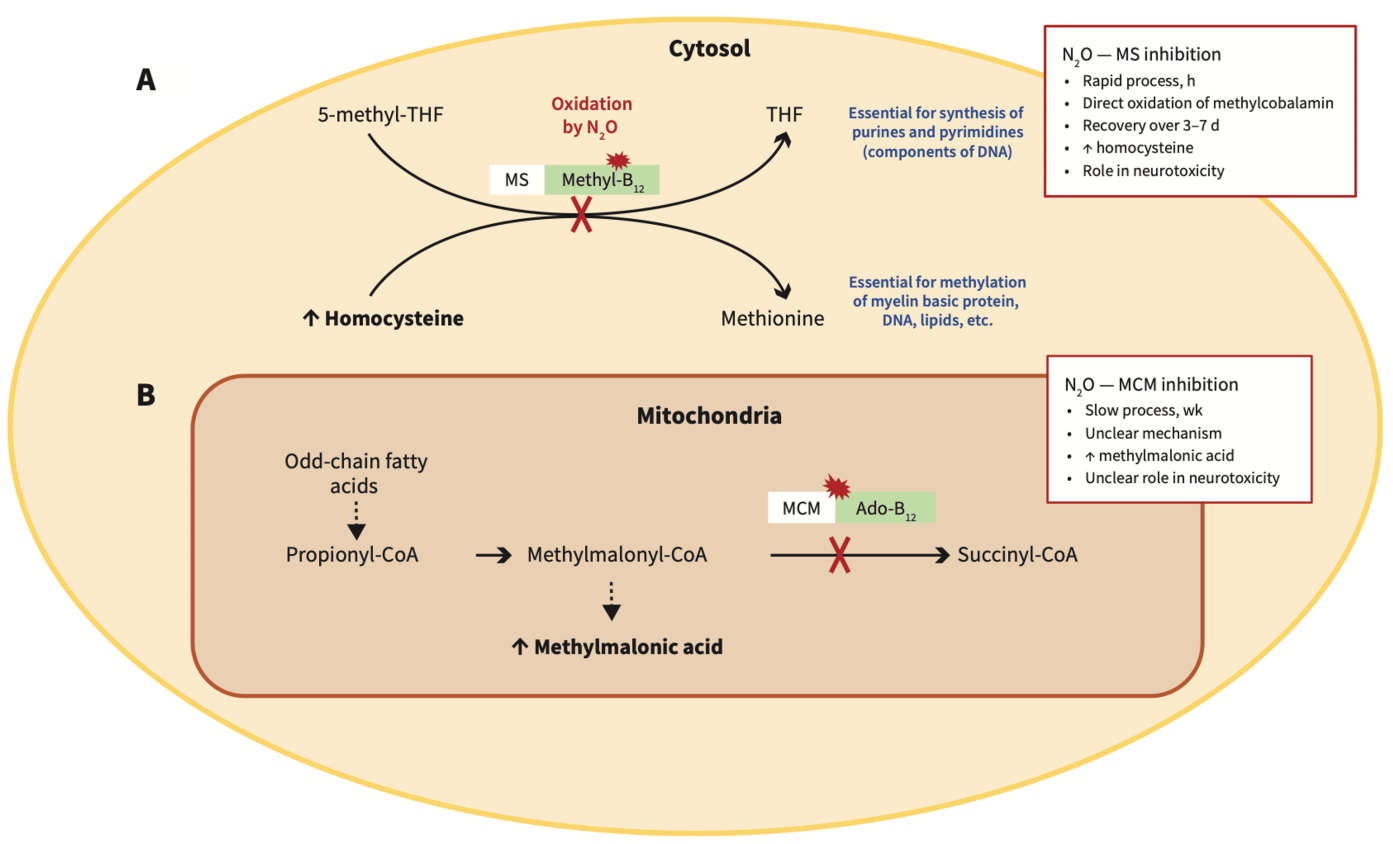
Interaction between nitrous oxide and vitamin B12 metabolic functions. (A) Methionine synthase (MS) converts homocysteine to methionine, and 5-methyltetrahydrofolate (methyl-THF) to tetrahydrofolate (THF). Methylcobalamin (the methyl form of vitamin B12) is an essential coenzyme in this process. Nitrous oxide (N_2_O) quickly and irreversibly oxidises the cobalt atom of methylcobalamin, rendering it inactive. The resulting inhibition of MS impairs folate activity and MS, which are essential for DNA production and myelin integrity. It also leads to an increase in homocysteine. MS inhibition largely underlies the neurotoxicity of N_2_O. (B) Methylmalonyl-CoA mutase (MCM) converts methylmalonyl-CoA to succinyl-CoA, which then enters the Krebs cycle. Adenosylcobalamin (Ado-B12; the adenosyl form of vitamin B12) is a cofactor for MCM. Prolonged use of N_2_O impairs the activity of MCM by unclear mechanisms. This increases methylmalonic acid concentration, which can be measured as a biomarker in patients with neurotoxicity secondary to N_2_O use. Note: White boxes = enzymes; green boxes = coenzymes of vitamin B12; red jagged circles = inhibition of enzyme activity; red “X” = interruption of the metabolic pathway. Image credit: De Halleux and Juurlink (2023) [2], under CC BY-NC-ND 4.0 licence.

However, N_2_O also has a significant metabolic impact. It irreversibly oxidises the cobalt ion in vitamin B12 (cobalamin), rendering it inactive. This inactivation inhibits methionine synthase, an enzyme critical for the remethylation of homocysteine to methionine, and for DNA and myelin synthesis [2,4]. Functional B12 deficiency may occur even in individuals with normal serum levels. The resulting accumulation of homocysteine and methylmalonic acid (MMA) contributes to neurological deficits and a pro-thrombotic state, manifesting as SCD of the spinal cord, peripheral neuropathy, and increased thromboembolic risk [5]. The interaction between N_2_O and vitamin B12 metabolic functions is shown in Figure 1.

Another key pathophysiological concern is cryogenic injury. Inhalation of N_2_O directly from high-pressure canisters or cartridges can lead to extreme cooling during gas expansion (Joule-Thomson effect), causing frostbite-like cold burns to the lips, oral cavity, pharynx, and upper airway. This can result in mucosal blistering, airway oedema, and even thermal necrosis, which may complicate airway management and intubation [6].

Neurological and haematological complications

The neurological effects of recreational N_2_O misuse are increasingly recognised and represent one of the most concerning long-term complications. These effects stem largely from functional vitamin B12 deficiency caused by irreversible inactivation of cobalamin by N_2_O. The enzyme methionine synthase becomes non-functional, leading to a block in DNA synthesis, myelin sheath disruption, and neuronal damage - even in patients without overt dietary B12 deficiency or anaemia [7]. Clinically, patients may present with paraesthesias and numbness, particularly in the hands and feet, along with an unsteady gait and ataxia due to posterior column involvement. Spastic paraparesis may occur, and in more severe cases, cognitive changes or psychosis can be observed. 

**Figure 2:**
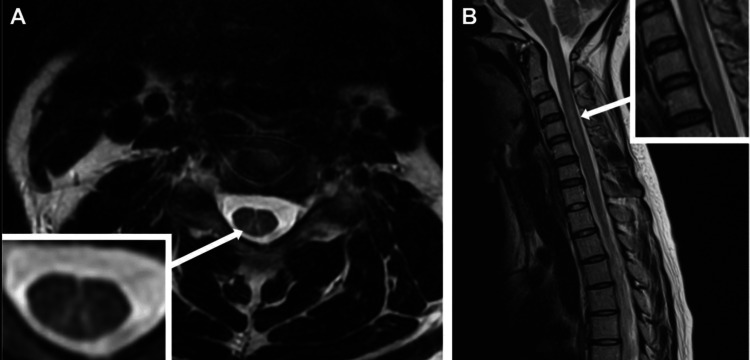
Typical MRI features of subacute combined degeneration of the spinal cord associated with nitrous oxide misuse. Axial T2-weighted image (A) reveals a hyperintense signal within the dorsal columns of the cervical spine, forming the characteristic “inverted V” pattern (arrow). Sagittal T2-weighted image (B) demonstrates longitudinally extensive dorsal column hyperintensity, spanning multiple cervical segments (arrow). Image credit: Paris et al. (2023) [8], under CC BY-NC 4.0 license. MRI, Magnetic resonance imaging

Magnetic resonance imaging (MRI) of the spinal cord typically shows T2 hyperintensities in the dorsal columns, especially in the cervical and upper thoracic regions, consistent with SCD (Figure 2) [8]. Nerve conduction studies may show axonal or demyelinating features.

In addition, haematological changes such as macrocytic anaemia, leukopenia, and thrombocytopenia may occur, although these are less frequent in acute settings [9]. Elevated MMA and homocysteine levels are more reliable markers of functional B12 deficiency than serum B12 itself, and they should be checked when neurological symptoms are suspected [10].

Prompt recognition is essential because early treatment with high-dose intramuscular hydroxocobalamin can reverse many of the neurological deficits. However, delayed diagnosis may result in permanent disability, especially with repeated exposure.

Airway and perioperative anaesthetic challenges

Recreational N_2_O use introduces several critical considerations for anaesthetists, particularly in emergency or perioperative contexts. These range from structural airway injury due to cryogenic trauma to delayed emergence, increased perioperative risk, and difficult airway scenarios [11].

Cryogenic Injury and Airway Burns

**Figure 3:**
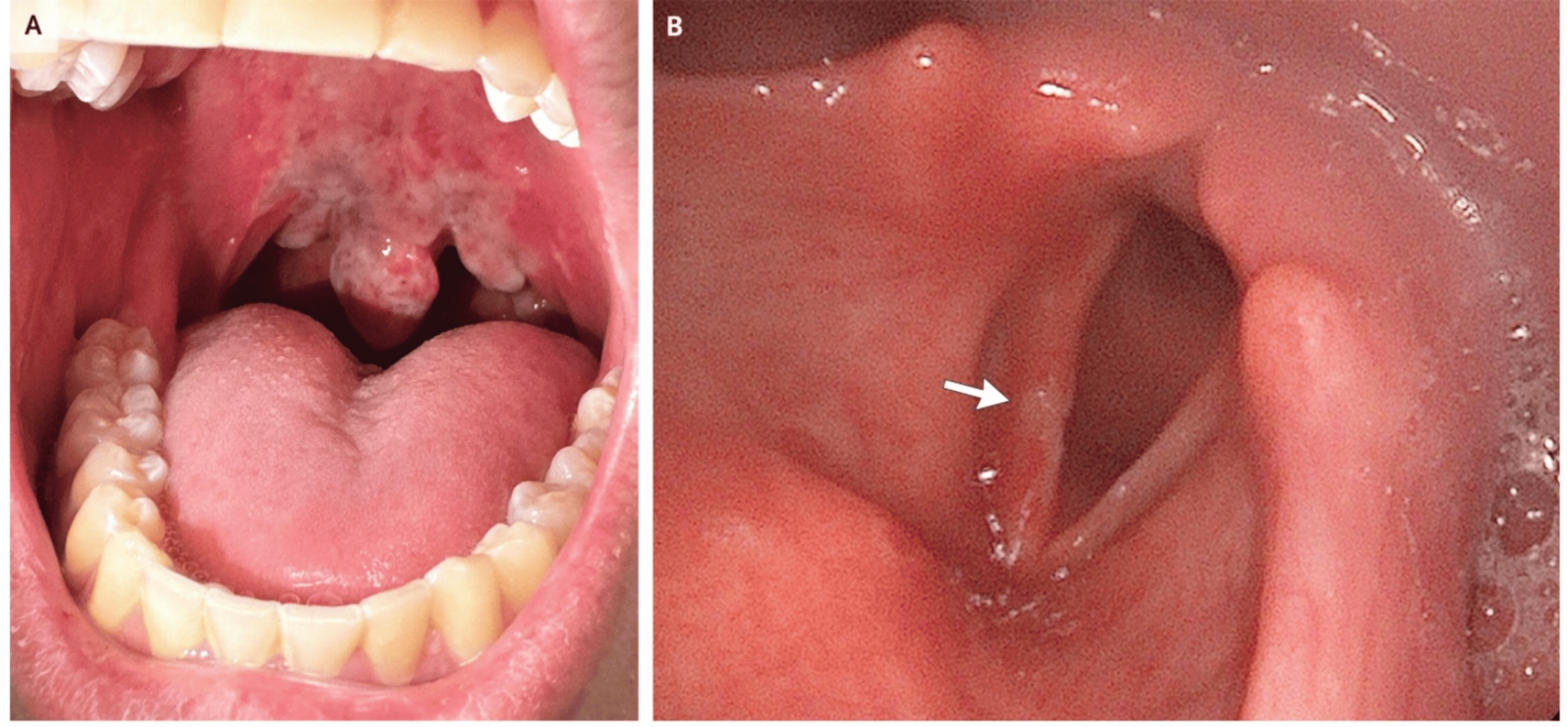
This image highlights upper airway mucosal injury from recreational nitrous oxide use, underscoring the importance of a thorough perioperative airway assessment. Panel (A) demonstrates marked erythema, swelling, and mucosal sloughing of the soft palate, uvula, and posterior oropharynx. Panel (B), obtained via flexible nasolaryngoscopy, shows a focal area of ulceration and oedema on the right vocal cord, highlighting potential laryngeal injury. Image credit: Reproduced with permission from Patrizio and Hayden (2025) [14].

Direct inhalation of pressurised N_2_O can result in cryogenic injury due to the Joule-Thomson effect, the rapid cooling that occurs when compressed gas expands, leading to tissue freezing. This may cause cold burns and frostbite-like injuries to the lips, tongue, oropharynx, and larynx, as well as oedema of the supraglottic and subglottic tissues. Mucosal necrosis and delayed sloughing have also been reported. These injuries can significantly complicate airway management, making mask ventilation or tracheal intubation difficult due to tissue distortion, bleeding, or oedema [12]. In such cases, clinicians may need to delay intubation, utilise video laryngoscopy for improved visualisation, and be prepared for surgical airway access if obstruction worsens. Early administration of corticosteroids, humidified oxygen, and vigilant airway monitoring is recommended to mitigate complications and maintain airway patency [13]. Figure 3 effectively illustrates the extent of upper airway involvement.

Preoperative Assessment and Risk Stratification

A comprehensive preoperative assessment should include a targeted history to identify N_2_O misuse, as patients may not disclose such use unless directly questioned. Clinical indicators that may suggest recreational N_2_O use include young age with unexplained neurological symptoms, such as ataxia or paraesthesias; macrocytic anaemia with elevated mean corpuscular volume (MCV) in the absence of other causes; or a history suggestive of frequent inhalation from “balloons” or whipped cream canisters. If misuse is suspected or confirmed, anaesthetists should perform a focused evaluation for airway-related signs, such as hoarseness, dysphagia, or mucosal burns; neurological abnormalities, including gait disturbances and impaired proprioception; and cardiovascular risks associated with elevated homocysteine levels. Additionally, the potential for delayed emergence from anaesthesia due to underlying neurological dysfunction must be considered [15-17].

Anaesthetic Considerations

Anaesthetic management of patients with a history of recreational N_2_O use should prioritise avoidance of further N_2_O exposure, as its continued administration may exacerbate functional vitamin B12 inactivation and associated complications. Total intravenous anaesthesia (TIVA) or volatile agents without N_2_O as a carrier should be used instead. Early establishment of intravenous access is recommended, and in patients displaying signs of airway oedema, such as hoarseness or dysphagia, awake intubation should be considered to ensure safe airway management [18].

Postoperative monitoring should be extended if there are concerns about respiratory compromise or neurological deterioration. A multidisciplinary approach is essential, with input from neurology, ear, nose, and throat (ENT) specialists, and addiction services, depending on the nature and severity of the patient’s presentation.

Recommendations for management

Based on current literature and clinical experience, several key strategies are recommended when managing patients with a history of recreational N_2_O use. A thorough substance use history should be incorporated into the preoperative anaesthetic assessment, as patients may not readily disclose N_2_O misuse without specific inquiry. A baseline neurological examination, focusing on gait, proprioception, and signs of sensory or motor deficits, is essential.

Laboratory investigations should include serum vitamin B12, MMA, homocysteine, and a full blood count to detect functional B12 deficiency or haematological abnormalities. If neurological symptoms are evident, a spinal MRI is warranted to assess for SCD. N_2_O should be excluded from the anaesthetic plan in all such cases. Where indicated, vitamin B12 supplementation should be initiated preoperatively. Additionally, detailed airway assessment is crucial, especially in recent users, with appropriate backup plans in place to manage potential cryogenic airway injuries or distortion.

## Conclusions

Recreational N_2_O misuse presents unique and potentially serious challenges to anaesthetists. While often perceived as a benign substance, N_2_O can cause profound neurological, haematological, and airway complications. Understanding the mechanisms of toxicity, recognising early signs of injury, and adapting anaesthetic management strategies are essential to reducing perioperative risk. As use becomes more prevalent, particularly in the UK and Europe, heightened vigilance and further education in the anaesthetic community are warranted.
